# Development of an improved competitive ELISA based on a monoclonal antibody against lipopolysaccharide for the detection of bovine brucellosis

**DOI:** 10.1186/s12917-015-0436-3

**Published:** 2015-05-21

**Authors:** Xiaolei Wang, Yan Wang, Limei Ma, Ran Zhang, Yanyan De, Xiaowen Yang, Chuanqing Wang, Qingmin Wu

**Affiliations:** Key Laboratory of Animal Epidemiology and Zoonosis of the Ministry of Agriculture, College of Veterinary Medicine, China Agricultural University, Beijing, China; Animal Infectious Disease Laboratory, College of Animal Science and Veterinary Medicine, Henan Agricultural University, Zhengzhou, China; Zhumadian Animal Disease Control Center, Zhumadian, China

**Keywords:** Brucellosis, Lipopolysaccharide, Monoclonal antibody, cELISA, Specificity

## Abstract

**Background:**

Brucellosis is the most common bacterial zoonosis, and serological tests are routinely used in brucellosis control and eradication programs. In order to improve the accuracy of serological diagnostic method used in bovine brucellosis detection, this study developed an improved competitive ELISA with higher specificity and good sensitivity.

**Results:**

This study prepared 12 monoclonal antibodies against smooth *Brucella* lipopolysaccharide. One monoclonal antibody 3 F9, presented C epitope specificity, was used to develop a competitive ELISA for the serological detection of bovine brucellosis. The competitive ELISA, a commercial competitive ELISA kit, the rose-bengal plate agglutination test, and a microplate agglutination test were all used in the detection of 6 hyperimmune antisera against other commonly cross-reacted bacterial pathogens and 110 clinical bovine serum samples. The results of the test comparisons indicated that the competitive ELISA had higher specificity than the commercial competitive ELISA kit and RBT, and comparable sensitivity with the commercial ELISA kit.

**Conclusions:**

This study provided a valuable detection tool with high specificity and good sensitivity, which prevent the wrong-culling of bovines in the eradication campaigns of bovine brucellosis.

**Electronic supplementary material:**

The online version of this article (doi:10.1186/s12917-015-0436-3) contains supplementary material, which is available to authorized users.

## Background

Brucellosis is the most common bacterial zoonosis caused by members of the *Brucella* genus, which infect a wide range of mammals, including dogs, ruminants, humans, and marine mammals*.* Within the last few years, brucellosis has re-emerged, presenting severe public health challenges and major economic burdens globally [1]. The measures to eradicate and control brucellosis outbreaks are principally based on an intensive test-and-slaughter policy [2, 3], in which effective technology to diagnose brucellosis plays an important role. Although bacterial isolation and identification of *Brucella* spp. is defined as the ‘gold standard’ for diagnosis of brucellosis, serological tests are routinely used in brucellosis control and eradication programs. Currently, the common serological diagnosis methods for bovine brucellosis include the serum agglutination test (SAT), the rose-bengal plate agglutination test (RBT), the milk ring test (MRT) [4, 5], the complement fixation test (CFT) [6], and primary binding assays such as the indirect ELISA (iELISA) [7, 8], the competitive ELISA (cELISA) [9, 10], and the fluorescence polarization assay (FPA) [11]. The majority of serological tests mentioned rely on the detection of antibodies against lipopolysaccharide (LPS). However, false positive results often occur from cross-reaction in the serological detection [12, 13], due to common antigens on LPS of *Brucella* and certain bacteria, especially *Yersinia enterocolitica* O:9 and *Escherichia coli* O157 [14, 15]. The sensitivity and specificity of different serological tests are variant [16]. Agglutination tests often do not have very good specificity. The CFT with high specificity and sensitivity has been approved, but tedious operations make it difficult to use for large-scale detection. In the past few decades, the FPA and iELISA with high sensitivity have been used for the diagnosis of brucellosis. The iELISA methods based on LPS antigens easily produce cross-reaction with the antibodies against other bacterial pathogens, which may result in over-culled animals. Unfortunately, the sensitivity of iELISA with protein antigens is not as good as the sensitivity of iELISA utilizing LPS [17, 18]. The FPA performs excellently for diagnosis but requires expensive specialized apparatus for measurement. These faults indicate that a high-throughput diagnostic methods with good specificity and sensitivity is necessary. The cELISA has become a reliable alternate diagnosis for brucellosis. However, of the limited sensitivity and specificity, the various monoclonal antibodies (MAbs) used in cELISA may result in omission or false detection in practical application. Therefore, an optimal cELISA for the diagnosis of animal brucellosis should be based on the MAb with high specificity and satisfactory properties.

LPS is a major surface antigen of *Brucella* that can be divided into smooth type (S) or rough type (R) depending on the inclusion or lack of O-polysaccharide (OPS) moiety. Four types of epitopes on the *Brucella* OPS have been described: the M and A epitopes, present on M and A dominant *Brucella* strains, respectively; the common (C) epitope, strictly specific for smooth *Brucella* spp., either A or M dominant; and the C/Y epitope, which is common to smooth *Brucella* spp. and *Y. enterocolitica* O:9 [19]. Different OPS epitopes are probably overlapping structures, but the C epitopes would be important to establish cELISA for the diagnosis of brucellosis.

For the serological detection of *Brucella*-infected cattle, 12 MAbs against smooth *Brucella* LPS were produced and characterized. Fortunately, among them, one was identified to be against C epitope. This MAb was selected to develop a competitive ELISA, which was compared with other methods for the detection of *Brucella* infection in cattle. The results showed that the developed cELISA demonstrated significantly improved specificity and good sensitivity.

## Results

### Screening and characterization of MAb

**Fig. 1:**
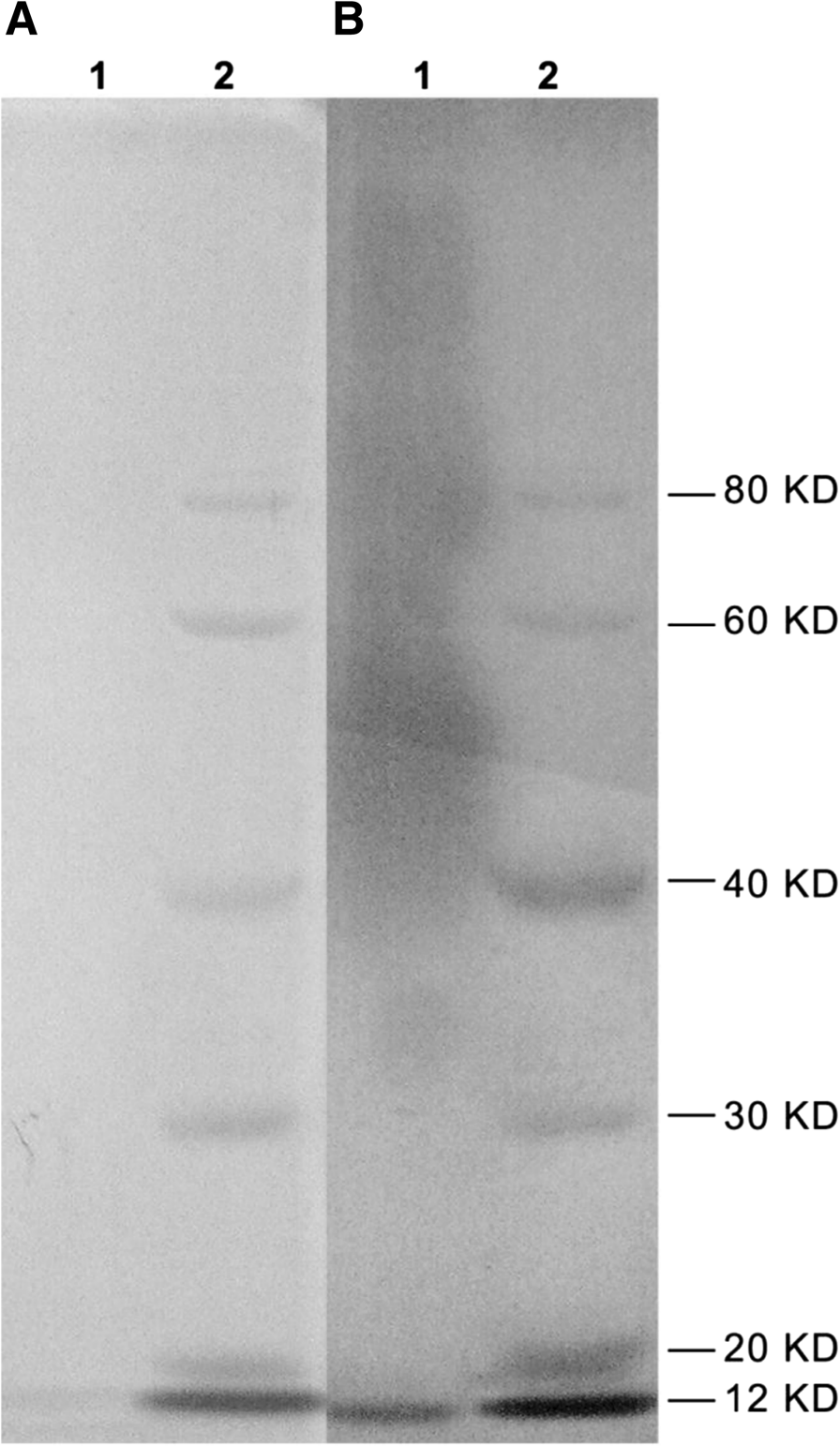
Coomassie blue-staining (**A**) and silver-staining (**B**) of LPS extracted from *B.melitensis* 16 M. LPS was prepared by hot phenol-water extraction method and fractionated by SDS-PAGE electrophoresis, followed by commassie blue (**A**) or silver (**B**) staining. LPS banding is seen (**B**). The absence of band in commassie blue staining as shown in A indicates no contamination of purified LPS with bacterial proteins. Lane 1: LPS, Lane 2: Molecular weight marker

This study immunized mice with heat-killed *B. melitensis* 16 M and boosted with large dose of purified LPS (Fig. 1). After four times of cell fusion, hybridomas screened by the iELISA established, twelve positive clones specific to LPS were obtained and then subcloned three times through the use of a limited dilution method. Among these MAbs, three were IgG1 (II5G1, 6E3, 4C3), four were IgG3 (3H7, 3 F9, 2C3, II4D11), four were IgM (6B8, 6 F2, 4H7, I2C10), and one was IgG2a.

### Characterization of specificity and epitope of MAb

**Fig. 2:**
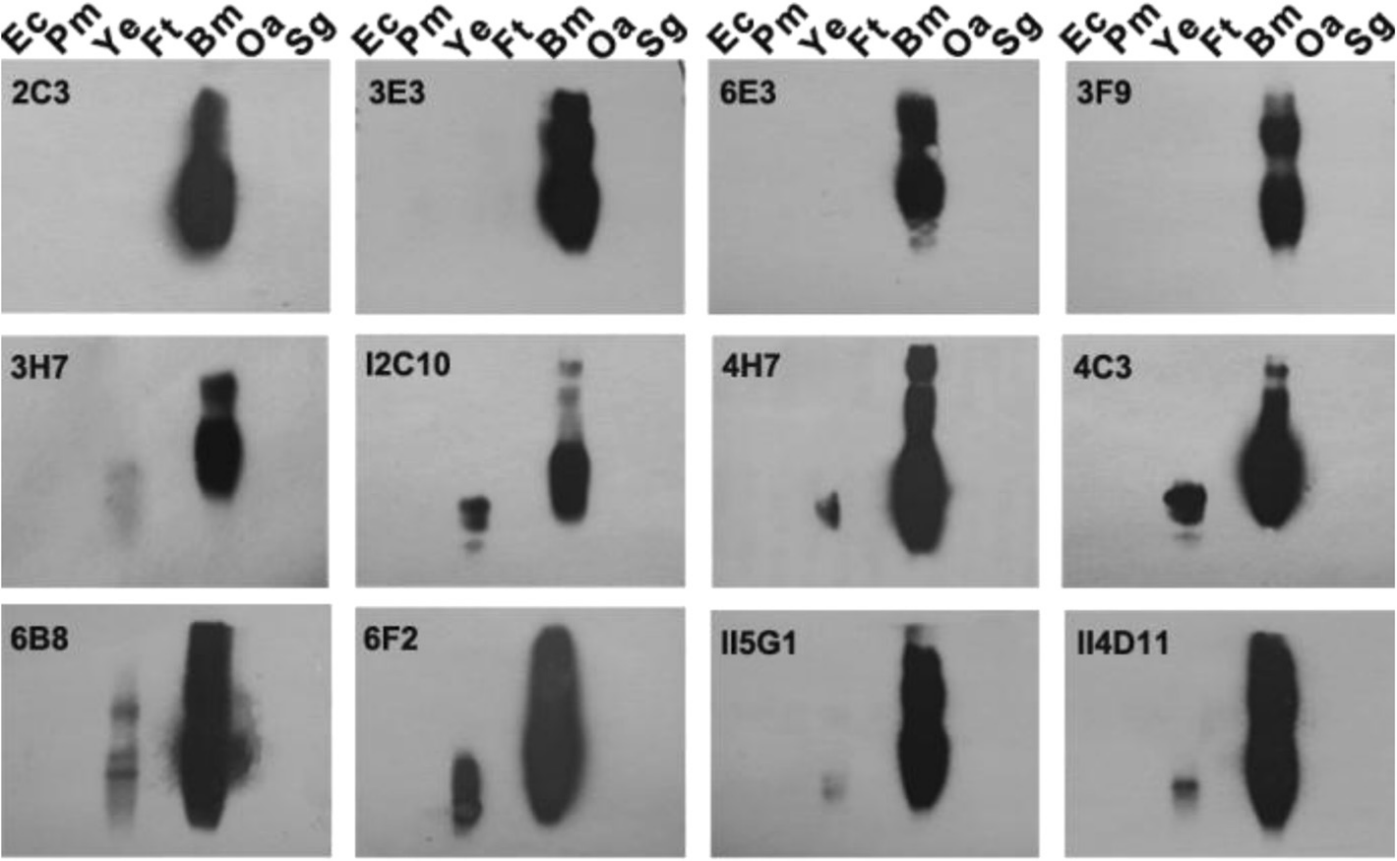
Western blot analysis for specifity of MAbs. Specificity of MAbs was investigated with whole-cell extracts of *Brucella melitensis* 16 M and different pathogens. Western blot were carried out as described in Materials and Methods. Abbreviation: *Ec: E. coli* O157; *Pm: P. multocida*; *Ye: Y. enterocolitica* O:9; *Ft: F. tularensis* LVS; *Bm: B. melitensis* 16 M; *Oa: O. anthropic* ATCC49188; *Sg: S. gallinarum*

In this study, the western blot with whole-cell lysates showed that four MAbs (2C3, 3E3, 3 F9, 6E3) were specific to *B.melitensis* 16 M. Meanwhile, the other eight MAbs (4H7, 4C3, 6B8, 6 F2, II5G1, II4D11, 3H7, I2C10) recognized epitope C/Y because they had weak cross-reactivity with *Y. enterocolitica* O:9 (Fig. 2). The results from the ELISA were consistent with the western blot test (Table 1).

**Table 1:** Indirect ELISA results for hybridoma supernatants

MAb	Ig class	OD^a^										Epitope recognized
		*Y. enterocolitica* O:9	*E. coli* O157	*S. gallinarum*	*O.* *anthropi*	*F.* *tularensis*	*P.* *multocida*	*RM*6/66(R)	NI (A^+^M^+^)	16 M (A^−^M^+^)	2308 (A^+^M^−^)	
2C3	IgG3	0.043	0.038	0.027	0.035	0.030	0.033	0.044	1.176	0.856	0.040	M
3E3	IgG1	0.037	0.053	0.036	0.039	0.031	0.042	0.041	1.419	0.942	0.044	M
6E3	IgG1	0.040	0.026	0.023	0.028	0.025	0.024	0.031	1.086	0.907	0.060	M
3H7	IgG3	0.350	0.061	0.046	0.045	0.043	0.057	0.035	1.115	0.567	1.544	C/Y(A > M)
3 F9	IgG3	0.037	0.053	0.031	0.042	0.037	0.049	0.034	1.022	0.983	0.514	C(M > A)
4C3	IgG2a	0.422	0.031	0.028	0.033	0.027	0.030	0.043	0.647	0.687	0.605	C/Y(M = A)
II5G1	IgG1	0.434	0.063	0.038	0.048	0.041	0.045	0.034	0.906	0.882	1.113	C/Y(M = A)
II4D11	IgG3	0.557	0.043	0.034	0.041	0.031	0.042	0.032	0.938	0.930	0.899	C/Y(M = A)
6B8	IgM	1.039	0.037	0.030	0.036	0.032	0.039	0.205	0.837	0.913	0.991	C/Y(M = A)
6 F2	IgM	0.959	0.023	0.021	0.033	0.031	0.024	0.048	0.897	0.899	0.845	C/Y(M = A)
4H7	IgM	0.442	0.030	0.028	0.035	0.030	0.034	0.043	0.946	0.853	0.898	C/Y(M = A)
I2C10	IgM	0.503	0.058	0.033	0.040	0.028	0.044	0.040	0.896	0.689	1.317	C/Y(A > M)

^a^OD of hybridoma supernatants in dilutions of OD 1.0 in iELISA with LPS of *B.melitensis* 16 M

**Fig. 3:**
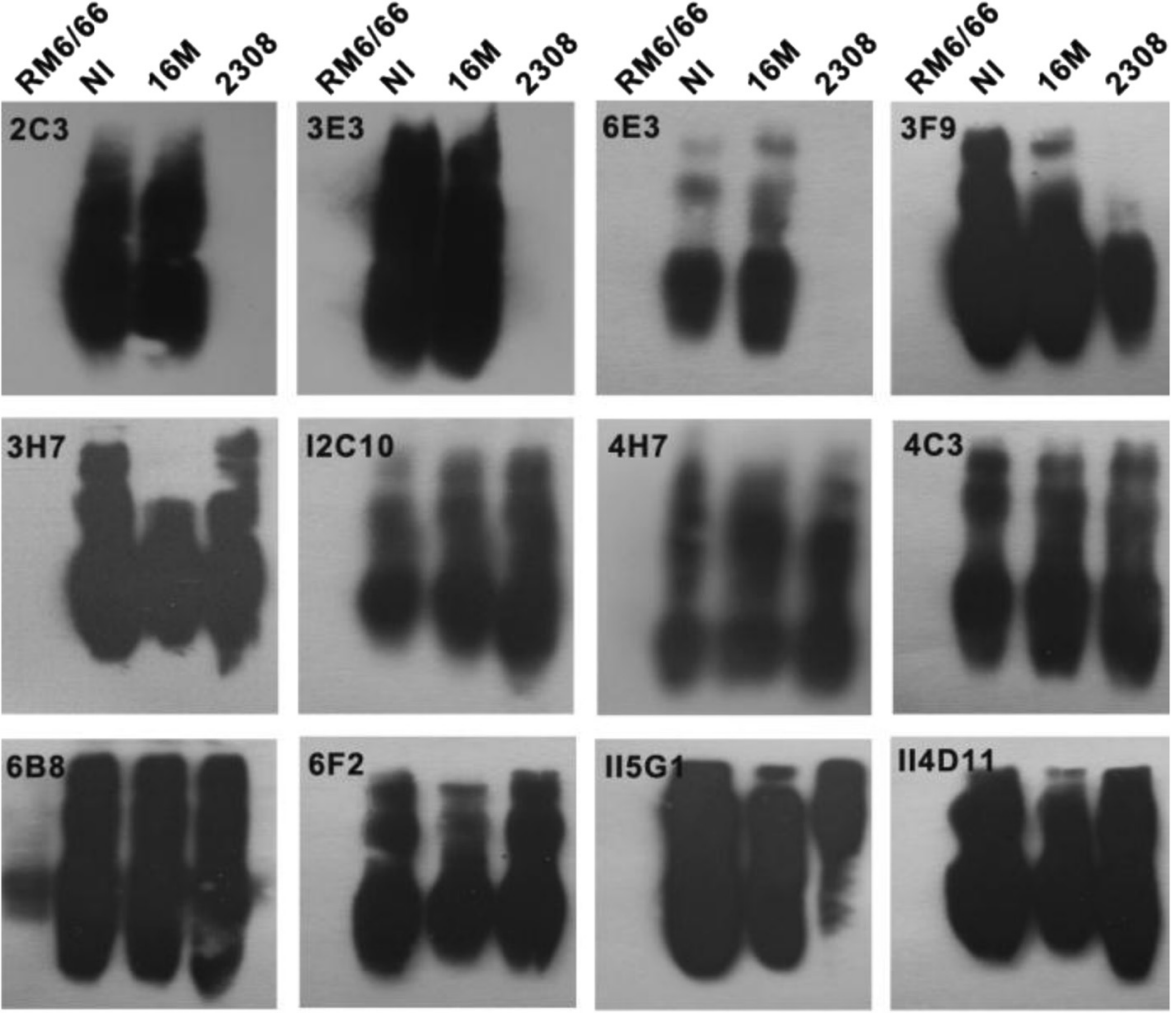
Western blot analysis for epitope of MAbs. Whole-cell extracts of *B. canis* RM6/66 (native rough type), *B. melitensis* NI (smooth type, A^+^M^+^), *B. melitensis* 16 M (smooth type, M-dominant, A^−^M^+^), *B. abortus* 2308 (smooth type, A-dominant, A^+^M^−^) were used to analyze the epitopic specificity of MAbs. SDS-PAGE and Western blot were carried out as described in Materials and Methods

C epitopes have been subdivided and seven epitopes on the Brucella OPS have been defined, including: A, M, C (M > A), C (A = M), C/Y (M > A), C/Y (A = M), and C/Y (A > M) [20, 21]. Further analysis of the MAb epitope specificity was performed with native rough phenotype *Brucella* and with smooth *Brucella* strains of three serotypes, i.e., A^+^M^−^, A^−^M^+^, and A^+^M^+^, corresponding to strains expressing mainly the A (A-dominant) or M (M-dominant) antigen or both antigens in nearly equivalent amounts [22] (Fig. 3). All the MAbs expect MAb 6B8, had no reactivity with rough type *B. canis* RM 6/66. MAb 2C3, 3E3, 6E3 reacted only with *B. melitensis* 16 M ((M-dominant, A^−^M^+^), which confirmed the specificity for M epitope. MAb 3 F9 was thought to be specific for the C epitope, as it bound to *B. melitensis* 16 M, *B. abortus* 2308 (A-dominant, A^+^M^−^), and *B. melitensis* NI (A^+^M^+^).

iELISA was performed to measure the MAbs relative binding level (Table 1). MAb 3 F9 bound equally to *B. melitensis* 16 M and *B. melitensis* NI, but had a weaker bind to *B. abortus* 2308, and thus, it recognized C (M > A) epitope. Aside from binding to *Y. enterocolitica* O:9, the binding level of MAbs 4H7, 4C3, 6B8, 6 F2, II5G1, II4D11 were generally equal relative to A or M dominance. These results confirmed their C/Y (A = M) specificity. As MAb 3H7, I2C10 bound to the A-dominant strain significantly stronger than to the M-dominant strain, they were thought to be specific for the C/Y (A > M) epitope.

### Determination of cut-off value and specificity of cELISA

**Fig. 4:**
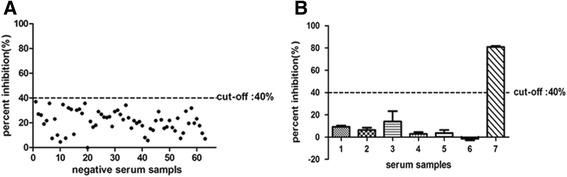
Establishment of the cELISA. (**A**) Percent inhibition values of 63 negative serum samples by cELISA. A cut-off value was set at 40 % (mean + 2SD). (**B**) Percent inhibition of polyclonal antisera against various bacteria serological related with smooth *Brucella*. 1 *Y. enterocolitica* O:9; 2 *E. coli* O157; 3 *S. gallinarum*; 4 *O. anthropic* ATCC49188; 5 *F. tularensis* LVS; 6 *P. multocida*; 7 positive bovine sera. Only the positive bovine sera had a PI exceeding the cut-off value. The Error bars indicate the standard deviations from three-well replications for each serum sample

On account of its specificity for C epitope, MAb 3 F9 was selected to develop a cELISA. After protocol optimization of blocking solution, dilution ratio of MAb and sample respectively, 63 negative bovine serum samples were used to determine the cut-off value. As the p-value of negative sera PI analyzed with the Shapiro-Wilk test was 0.482, this paper concluded that the data were distributed normally. The mean PI value was 22 % (SD = 9 %) for the bovine. Therefore, the cut-off value (mean + 2SD) to determine the status of the serum samples in response to smooth *Brucella* was set at 40 % (Fig. 4A).

To confirm its specificity, the cELISA was used to detect the polyclonal antisera against *E. coli* O157, *S. gallinarum*, *P. multocida*, *Y. enterocolitica* O:9, *F. tularensis LVS*, and *O. anthropic* ATCC49188. The results showed that the PI values of all of these sera were much lower than the cut-off value (Fig. 4B).

### Comparison among cELISA,a commercial cELISA kit, RBT and MAT

Six hyperimmune antisera and 110 clinical bovine serum samples were tested with the cELISA. The resulting data were compared with data obtained from a commercial cELISA kit, the RBT and the MAT. Five of the six hyperimmune antisera were negative in the four diagnostic tests. Notably, the antisera of *Y. enterocolitica* O:9 detected as negative in the cELISA and the MAT, but as positive in the commercial kit and the RBT (Table 2).

**Table 2:** Results of 6 hyperimmune antisera detection by cELISA, commercial kit, RBT and MAT

Sample name	cELISA	Commercial kit	RBT	MAT
*E. coli* O157	**-**	**-**	**-**	**-**
*O. anthropic*	**-**	**-**	**-**	**-**
*F. tularensis* LVS	**-**	**-**	**-**	**-**
*S. gallinarum*	**-**	**-**	**-**	**-**
*Y. enterocolitica* O:9	**-**	**+**	**+**	**-**
*P. multocida*	**-**	**-**	**-**	**-**

-: negative result, +: positive result

Among the bovine sera, the positive rate detected by the cELISA was 54.5 % (60/110), compared to 48.2 % (53/110), 62.7 % (69/110), and 42.7 % (47/110) by the commercial kit, RBT, and MAT, respectively. Data appear in Table 3, by all four methods, 40 sera were tested negative and 43 sera tested positive. One sample tested positive in the commercial kit but negative in the other three tests. The additional 26 serum samples tested positive with RBT, and of those sera, 17 were confirmed positive with the cELISA, while the commercial kit and the MAT missed these positive sera (5 were missed by both the commercial kit and the MAT, 4 by just the commercial kit, and 8 by just the MAT). Eight sera were negative, with the exception of the RBT, and one was negative in the cELISA and the MAT, but positive in the commercial kit and RBT. The cELISA agreed well with the commercial kit (kappa = 0.801), the RBT (kappa = 0.832), and the MAT (kappa = 0.767). The supplement shows detail detection results of all of the serum samples (Additional file 1: Table S1).

**Table 3:** Results of 110 bovine sera samples for *Brucella* antibody detection by cELISA, commercial kit, RBT and MAT

Sample number	cELISA	commercial kit	RBT	MAT
43	**+**	**+**	**+**	**+**
40	**-**	**-**	**-**	**-**
8	**+**	**+**	**+**	**-**
8	**-**	**-**	**+**	**-**
5	**+**	**-**	**+**	**-**
4	+	-	+	+
1	**-**	**+**	**+**	**-**
1	**-**	**+**	**-**	**-**

-: negative result, +: positive result

## Discussion

Domestic animals infected with *Brucella spp*. are culled partly based on the detection of the antibodies against *Brucella* LPS antigen. However, other bacterial infections often disturb the serological examination of brucellosis [12, 13], due to the antigens of cross-reaction among the bacteria. It is known that smooth *Brucella* LPS contains O-polysaccharide (OPS) moiety, which has been divided into seven epitopic specificities: A, M, C (M > A), C (A = M), C/Y (M > A), C/Y (A = M), and C/Y (A > M) [20, 21]. Research has shown that the cross-reactions in the brucellosis serological detection mainly occur as a result of the similar structures that A and C/Y epitopes share with the OPS of *Y. enterocolitica* O:9 and other bacteria [14, 15]. These possible inaccuracies in serological examination methods due to cross-reaction may result in the wrong-culling of animals in the campaigns to eradicate animal brucellosis.

In this study, 12 MAbs against LPS of *Brucella melitensis* 16 M were characterized, among which four were IgM MAbs, four were IgG3, three were IgG1, and one was IgG2a. *Brucella* strains of three serotypes belonging to *Brucella melitensis* and *Brucella abortus* were used as representations to identify the epitope specificity of MAbs. *Brucella suis,* which possess five biovars of three serotypes were not used due to the lack of the strains in the laboratory. As *Brucella suis* biovar 2 displayed unique reactivity with MAbs of C (A = M) and C (M > A) [21], identification of MAbs with *Brucella suis* biovar 2 may provide some interesting results. Almost all of the Mabs were against the C/Y or M epitopes, with the exception of MAb 3 F9. Interestingly, among the six MAbs that recognized C/Y (A = M) epitope, MAb 6B8 reacted faintly with *B. canis* RM 6/66 (Table 1, Fig. 3), which led this study to analyze whether there were discrepancies between the epitopes of these MAbs. The ELISA additivity test [23] was applied to the six MAbs, and the results (see Additional file 1) showed that either two MAbs failed to bind simultaneously to the antigen. As the core oligosaccharide is common to rough and smooth LPS, MAb 6B8 likely differed from the others by recognizing this region rather than just the formamido-mannose polymer in OPS [24]. The epitope of MAb 6B8 necessitates further analysis.

Fortunately, one monoclonal antibody 3 F9, which belonged to the IgG3 isotype, was identified to be specific for C epitope as evidenced by western blot and iELISA in this study. MAb 3 F9 indicates good specificity, as it did not cross-react with *E. coli* O157, *S. gallinarum*, *P. multocida*, *Y. enterocolitica* O:9, *F. tularensis* LVS, or *O. anthropic* ATCC49188. Furthermore, the MAb 3 F9 was used to establish a successful cELISA for the examination of bovine brucellosis. As Cattle is mainly infected by *B. abortus*, sometimes can also be infected by *B.melitensis* and by *B. suis* when they share pastures or facilities with infected pigs, goats and sheep [25, 26], the method in this study would be extensively applicable because *Brucella* C epitopes appear in all smooth *Brucella* spp. Besides, this proposed method could eliminate the nonspecific serological reaction in the examination of bovine brucellosis.

The established cELISA was compared with RBT, MAT and a commercial cELISA kit for the detection of six hyperimmune antisera and 110 clinical bovine serum samples. The results showed that all of six hyperimmune antisera, including antisera against *E. coli* O157 and *Y. enterocolitica* O:9 that commonly cross-reacted in brucellosis serological diagnosis, presented negative responses in the cELISA test. However, the hyperimmune antisera against *Y. enterocolitica* O:9 tested positive in the commercial kit and RBT. MAT test determined its antibody titer to be 25. The results demonstrate that the cELISA established in this study possesses higher specificity than the commercial cELISA and RBT. Multiple serum samples from animals infected with these pathogens may be more suitable for evaluation of the specificity. However, in consideration of the hyperimmune antisera used in this test with higher titer and affinity than clinical sera, it’s persuasive for the good specificity of the cELISA.

The results of 110 clinical bovine sarum samples by the cELISA coincided with the results of the commercial kit and RBT at rates of 90 % (99/110), and 91.82 % (101/110), respectively. All the samples appearing negative in RBT test also tested negative in the improved cELISA. Moreover, the samples that had antibody titers over 200 in MAT test also had a positive response in the cELISA, the commercial kit, and RBT tests. These results shown in detail in an additional table (see Additional file 2) suggest that the cELISA has a good detection performance. Of samples positive in RBT test and of the antibody titers 50–100 in MAT test, 100 % (23/23) samples tested positive in the cELISA test, and only 69.6 % (16/23) tested positive in the commercial cELISA test. These results indicate that the cELISA may have better sensitivity in the detection of serological positive state with low antibody titer. A further accurate comparison may need more serum samples in status of low antibody titer. These comparative results also showed that RBT was the most sensitive and suitable for screening test in bovine brucellosis.

Although this preliminary results indicated the applicable prospects of the cELISA for the specific detection of bovine brucellosis, further evaluating and optimizing the detection performance of this method was indispensable in the future with the standardized positive and negative sera, and more clinical bovine serum samples. It was reported that cELISA could be used to distinguish vaccinated antibody [9, 16]. In this study the ability of differentiating cattle vaccinated with S19 from infected with *Brucella* field strains was also required for evaluation of the detection performance of the cELISA. Some *Brucella*-infected animals reacted negatively to serological tests [27, 28], which indicated an early-stage infection or the bacterial loads were too few. More research should be conducted on these cases.

## Conclusions

This study prepared twelve monoclonal antibodies against smooth *Brucella* lipopolysaccharides through cell fusions and then characterized them by western blot and iELISA. One monoclonal antibody 3 F9 presented the IgG3 subclass, C epitope specificity. Mab 3 F9 was used to establish a cELISA for the detection of bovine brucellosis. A comparison of the cELISA with a commercial cELISA kit, RBT and MAT showed that, the cELISA had more specificity than the commercial cELISA kit and RBT, and comparable sensitivity with the commercial cELISA kit. This study provided a valuable detection tool with higher specificity and good sensitivity, which prevent the wrong-culling of bovines in the eradication campaigns of bovine brucellosis.

## Methods

### Bacterial strains

The *B. abortus* 2308, *B. melitensis* 16 M, and *B. canis* RM6/66 were all kindly donated by Qianni He (Institute of Veterinary Research, Xinjiang Academy of Animal Sciences, China). These strains were originally collected and preserved in the China Veterinary Culture Collection Center (CVCC, Beijing, China). The epidemic strain *B. melitensis* NI was isolated from an aborted bovine fetus from Inner Mongolia by this laboratory. *Escherichia coli* O157 and *Salmonella gallinarum* were preserved in the laboratory and originally collected in CVCC. *Ochrobactrum anthropic* ATCC49188 was purchased from the Guangdong Microbiology Culture Center. The inactivated culture of *Yersinia enterocolitica* O:9 and *Francisella tularensis* Live Vaccine Strain were gifts from the Huaiqi Jing (National Institute for Communicable Disease Control and Prevention, Chinese Center for Disease Control and Prevention, Beijing, China) and the Jingliang Su (China Agricultural University, Beijing, China) respectively. *Pasteurella multocida* was isolated from the field and identified in this laboratory previously. *Brucella* strains and *O. anthropic* ATCC49188 were routinely grown in either tryptic soy broth (BD, USA) or tryptic soy agar. The *E. coli* O157 and the *S. gallinarum* were grown in Luria-Bertani, and the *P. multocida* was grown in Brain Heart Infusion Broth (BD, USA). All operations with living *Brucella* strains were performed in biosafety level 3 facilities at China Agricultural University.

### Serum samples

Negative bovine serum samples were collected from cattle without a history of *Brucella* infection. Clinical bovine serum samples were collected from the Beijing and Hebei provinece in China and stored at −20 °C.

Rabbit hyperimmune antisera against *E. coli* O157, *S. gallinarum*, *P. multocida*, *Y. enterocolitica* O:9, *F. tularensis* LVS, and *O. anthropic* ATCC49188 were all prepared previously with immunization with inactivated cultures four times every two weeks. The antisera were used for the specificity analysis of the established cELISA.

### Antigenic preparations

For mice immunization and MAb screening, LPS was prepared from *B. melitensis* 16 M by the hot phenol-water method [29] and then analyzed for purity by silver-staining and Coomassie Blue Staining [30]. The *Brucella* strains were heat-killed at 68 °C for 2 h, and the other bacteria were inactivated with 0.2 % formaldehyde. For MAb specificity and epitope analysis, all the bacteria were resuspended with PBS and adjusted to OD_600_ approximately 1.0. For western blot, cell lysates were obtained by boiling in the presence of SDS. For ELISA, whole-cell lysates were obtained by ultrasonication.

### Establishment of MAb cell lines

Five 4 to 6-week-old female BALB/c mice were purchased from the Jinmuyang Laboratory Animal Breeding Co., LTD (Beijing, China). All animal studies complied with the guidelines for laboratory animal welfare and ethics set forth by the Beijing Administration Committee of Laboratory Animals and were approved by the Animal Care and Use Committee of China Agricultural University. The mice were immunized with 10^8^ CFU heat-killed *B. melitensis* 16 M organisms by intraperitoneal injection. The mice were boosted with the same dose at Week 3 and Week 5. Blood was collected from the tail in order to check the antibody titer, and then the mouse producing the highest titer was selected for hybridoma production. Serial boosters using an intraperitoneal injection of 50 μg of *B. melitensis* 16 M LPS were given every day during the three days prior to the cell fusion to improve the positive rate of antibody secreting hybridoma. Spleen cells from immunized mice were fused with Sp2/0 mouse myeloma cells at a ratio of 5:1 in the presence of 50 % (w/v) PEG 4000. Then, the fused cells were plated into 96-well microplates in DMEM with HAT media supplemented with 20 % fetal bovine serum. The hybridoma cells were cultured in the HAT media for the first 7 days and then in the HT media for the next 7 days. The culture supernatants were screened for specific antibodies against LPS by iELISA as described below. The positive hybridoma clones were subcloned three times by limiting dilution until monoclones were obtained. The stable antibody-producing clones were expanded and cryopreserved in liquid nitrogen. MAb culture supernatants were produced and collected as described previously [31].

### iELISA for screening MAb

The 96-well microplates were coated with LPS (0.5 μg/ml, 100 μl/well in 0.05 M carbonate buffer, pH 9.6) at 4 °C overnight. Nonspecific protein binding was blocked with 5 % skim milk in PBST (0.01 M, pH7.2 PBS containing 0.05 % Tween-20) at 37 °C for 30 min. Hybridoma supernatants and HRP-conjugated goat anti-mouse IgG (KPL, USA) diluted 1:5,000 in PBST were sequentially added and incubated at 37 °C for 1 h. Substrate solution containing 3,3′,5,5′-tetramethylbenzidine (TMB) was added to each well at 37 °C for 10 min for visualization. The reaction was stopped with 2 M sulfuric acid. An automatic ELISA plate reader (BioTek synergy ™ 2, USA) was used to measure the absorbance at 450 nm, and OD above 0.1 was consider positive.

### ELISA to determine the isotype of MAb

The isotype of the MAbs were determined by testing the hybridoma cell culture supernatants with a mouse monoclonal antibody isotyping kit (SBA, USA), according to the manufacturer’s instructions.

### Analysis for MAb specificity and epitope

In Western blot, whole-cell extracts of *Brucella* and other bacteria were resolved by 12 % SDS-PAGE and then transferred to a nitrocellulose membrane. The membrane was blocked with 10 % skim milk at room temperature for 3 h, then incubated with MAb supernatants at room temperature for 1 h. Unbounded antibody was washed off, and then the HRP-conjugated goat anti-mouse IgG was incubated on the membrane at room temperature for 50 min. Unbound conjugate was then washed off, and the membrane was added to ECL substrate, placed under an x-ray film, and exposed in a dark room.

In iELISA, 96-well microplates were incubated at room temperature overnight with whole-cell lysates 100 μl per well. After blocking, the MAb supernatants were added to each well, and the ELISA was conducted as described above.

### Establishment of cELISA

The microplates were coated with heat-killed whole-cell *B. melitensis* NI cultures in PBS (OD_600_ approximately 1.0) 10 μl/well, and then placed in an incubator at 40-50 °C until the liquid completely evaporated. Next, 5 % glutaraldehyde solution in 0.1 M sodium bicarbonate was added to fix the antigens and left at room temperature overnight. After blocking with 5 % skim milk at 37 °C for 1 h, 50 μl of the serum sample 1:20 diluted in blocking agents was added to each well, followed immediately by the addition of 50 μl of MAb 3 F9 supernatants 1:400 diluted in blocking agents. The plates were incubated at 37 °C for 1 h with shaking during the initial 3 min. Set up in duplicate wells, the controls included a strong positive, a weak positive, a negative control serum, and a buffer control. After washing with PBST, HRP-conjugated goat anti-mouse IgG 1:6000 diluted in PBST was added and incubated at 37 °C for 40 min. The unbound conjugates were removed by washing, and the chromogenic reaction was achieved as described above. As the bottoms of the plates were covered by the glutaraldehyde solution, two wavelength (450 nm and 630 nm) were used to measure the OD, i.e. OD_450_-OD_630_. The percent inhibition (PI) value was determined using the formula: PI (%) =100 – (OD [test sample] / OD [buffer control] × 100).

Sixty-three bovine serum samples from cattle without a history of *Brucella* infection, which were previously confirmed negative through the RBT and a microplate agglutination test (MAT), were used to determine the PI cut-off value that was designed as the mean PI of negative sera + 2 standard deviations (SD), in order to ensure that 95 % of PI values for the negative sera fell within this range.

To evaluate its specificity, the cELISA was used to detect polyclonal antisera against *E. coli* O157, *S. gallinarum*, *P. multocida*, *Y. enterocolitica* O:9, *F. tularensis* LVS, and *O. anthropic* ATCC49188. PI for these antisera were also calculated.

### Comparisons of cELISA, a commercial cELISA kit, RBT and MAT

The 6 polyclonal antisera mentioned above and 110 bovine serum samples were diagnosed using the cELISA, a commercial cELISA kit (SVANOVIR® *Brucella*-Ab C-ELISA, Sweden), RBT, and MAT. The ELISA kit was used in the manner as recommended by the manufacturer. The RBT was operated as previously described [22]. The MAT was performed as reported [32], but with some modification. Serum samples diluted 1:12.5 and 2-folded serially in the PBS were prepared in a 96-well V-bottom microplate. An equal volume (50 μL) of antigen solution was added to each well. The sealed plates were incubated in a humid atmosphere at 37 °C for 24 h. The titer of serum samples was expressed as reciprocal of the highest dilution of sera showing completely agglutination. The positive serum samples had titer 100 or more, and the suspicious serum samples had titer of 50. The smooth antigens used in the RBT and the MAT were purchased from the China Institute of Veterinary Drug Control.

### Statistical analysis

SPSS v20.0 software was used to analyze the result of a normality test of the negative sera PI values using a Shapiro-Wilk test. The software was also used to analyze the degree of agreement between the cELISA, a commercial kit, RBT, and MAT by kappa statistics.

## Additional files

Additional file 1:
**Epitope mapping of MAbs (C/Y(M = A)) on additivity test.** The additivity index (AI) value were calculated. The value below 50 indicated that the MAbs tested recognized the same epitope. Additional file 2:
**Detection results of four serological diagnosis.** The detail detection results of 110 bovine sera samples and 6 hyperimmune antisera in four serological diagnosis were shown in the table.

## Abbreviations

MAb: monoclonal antibody
RBT: the rose-bengal plate agglutination test
tCFT: he complement fixation test
FPA: the fluorescence polarization assay
MAT: microplate agglutination test
cELISA: competitive ELISA
iELISA: indirect ELISA
LPS: lipopolysaccharide
PI: percent inhibition

## References

[1] Seleem MN, Boyle SM, Sriranganathan N (2010). Brucellosis: A re-emerging zoonosis. Vet Microbiol.

[2] Martins H, Garin-Bastuji B, Lima F, Flor L, Pina Fonseca A, Boinas F (2009). Eradication of bovine brucellosis in the Azores, Portugal-Outcome of a 5-year programme (2002–2007) based on test-and-slaughter and RB51 vaccination. Prev Vet Med.

[3] Park SY, Kim TJ, Yoon H, Kim JY, Lee MJ, Lee WC (2012). A retrospective study of the extensive eradication program for brucellosis outbreaks and control in Korea, 2002–2009. Jpn J Infect Dis.

[4] Gurturk K, Boynukara B, Ilhan Z, Hakki Ekin I, Gulhan T (1999). Comparison of the dot-immunobinding assay with the serum agglutination test, the rose bengal plate test and the milk ring test for the detection of Brucella antibodies in bovine sera and milk. Zentralbl Veterinarmed B.

[5] Cadmus SIAH, Stack J (2008). The use of the milk ring test and rose bengal test in brucellosis control and eradication in Nigeria. J S Afr Vet Assoc.

[6] Mathias LA, Pinto AA (1983). Comparative study among complement fixation, serum agglutination and Rose Bengal Plate tests in the serodiagnosis of bovine brucellosis. Int J Zoonoses.

[7] Uzal FA, Carrasco AE, Echaide S, Nielsen K, Robles CA (1995). Evaluation of an indirect ELISA for the diagnosis of bovine brucellosis. J Vet Diagn Invest.

[8] Nielsen K, Smith P, Gall D, Perez B, Cosma C, Mueller P, Trottier J, Cote G, Boag L, Bosse J (1996). Development and validation of an indirect enzyme immunoassay for detection of antibody to Brucella abortus in milk. Vet Microbiol.

[9] Nielsen KH, Kelly L, Gall D, Nicoletti P, Kelly W (1995). Improved competitive enzyme immunoassay for the diagnosis of bovine brucellosis. Vet Immunol Immunopathol.

[10] Nielsen K, Smith P, Yu WL, Elmgren C, Halbert G, Nicoletti P, Perez B, Conde S, Samartino L, Nicola A (2008). Validation of a second generation competitive enzyme immunoassay (CELISA) for the diagnosis of brucellosis in various species of domestic animals. Vet Immunol Immunopathol.

[11] Nielsen K, Gall D, Lin M, Massangill C, Samartino L, Perez B, Coats M, Hennager S, Dajer A, Nicoletti P (1998). Diagnosis of bovine brucellosis using a homogeneous fluorescence polarization assay. Vet Immunol Immunopathol.

[12] Saegerman C, De Waele L, Gilson D, Godfroid J, Thiange P, Michel P, Limbourg B, Vo TK, Limet J, Letesson JJ (2004). Evaluation of three serum i-ELISAs using monoclonal antibodies and protein G as peroxidase conjugate for the diagnosis of bovine brucellosis. Vet Microbiol.

[13] Nielsen K, Smith P, Widdison J, Gall D, Kelly L, Kelly W, Nicoletti P (2004). Serological relationship between cattle exposed to *Brucella abortus, Yersinia enterocolitica* O:9 and *Escherichia coli* O157:H7. Vet Microbiol.

[14] Chart H, Okubadejo OA, Rowe B (1992). The serological relationship between *Escherichia coli* O157 and *Yersinia enterocolitica* O9 using sera from patients with brucellosis. Epidemiol Infect.

[15] Caroff M, Bundle DR, Perry MB (1984). Structure of the O-chain of the phenol-phase soluble cellular lipopolysaccharide of *Yersinia enterocolitica* serotype O:9. Eur J Biochem.

[16] Nielsen K (2002). Diagnosis of brucellosis by serology. Vet Microbiol.

[17] Letesson JJ, Tibor A, van Eynde G, Wansard V, Weynants V, Denoel P, Saman E (1997). Humoral immune responses of Brucella-infected cattle, sheep, and goats to eight purified recombinant *Brucella* proteins in an indirect enzyme-linked immunosorbent assay. Clin Diagn Lab Immunol.

[18] Xin T, Yang H, Wang N, Wang F, Zhao P, Wang H, Mao K, Zhu H, Ding J (2013). Limitations of the BP26 protein-based indirect enzyme-linked immunosorbent assay for diagnosis of Brucellosis. Clin Vaccine Immunol.

[19] Palmer DA, Douglas JT (1989). Analysis of *Brucella* lipopolysaccharide with specific and cross-reacting monoclonal antibodies. J Clin Microbiol.

[20] Weynants V, Gilson D, Cloeckaert A, Tibor A, Denoel PA, Godfroid F, Limet JN, Letesson JJ (1997). Characterization of smooth lipopolysaccharides and O polysaccharides of *Brucella* species by competition binding assays with monoclonal antibodies. Infect Immun.

[21] Cloeckaert A, Weynants V, Godfroid J, Verger JM, Grayon M, Zygmunt MS (1998). O-Polysaccharide epitopic heterogeneity at the surface of Brucella spp. studied by enzyme-linked immunosorbent assay and flow cytometry. Clin Diagn Lab Immunol.

[22] Alton GG, Jones LM, Angus RD, Verger JM (1988). Techniques for the Brucellosis Laboratory.

[23] Hu JQ, Li YF, Guo JQ, Shen HG, Zhang DY, Zhou JY (2007). Production and characterization of monoclonal antibodies to (poly100)S1 protein of avian infectious bronchitis virus. Zoonoses Public Health.

[24] Cardoso PG, Macedo GC, Azevedo V, Oliveira SC (2006). *Brucella* spp noncanonical LPS: structure, biosynthesis, and interaction with host immune system. Microb Cell Fact.

[25] Ewalt DR, Payeur JB, Rhyan JC, Geer PL (1997). *Brucella suis* biovar 1 in naturally infected cattle: a bacteriological, serological, and histological study. J Vet Diagn Invest.

[26] Kahler SC (2000). *Brucella melitensis* infection discovered in cattle for first time, goats also infected. J Am Vet Med Assoc.

[27] Zowghi E, Ebadi A, Mohseni B (1990). Isolation of *Brucella* organisms from the milk of seronegative cows. Rev Sci Tech.

[28] O'Grady D, Byrne W, Kelleher P, O'Callaghan H, Kenny K, Heneghan T, Power S, Egan J, Ryan F (2014). A comparative assessment of culture and serology in the diagnosis of brucellosis in dairy cattle. Vet J.

[29] OIE: Manual of Diagnostic Tests and Vaccines for Terrestrial Animals. http://www.oie.int/eng/normes/mmanual/A_summry.htm; 2009.

[30] Rezania S, Amirmozaffari N, Tabarraei B, Jeddi-Tehrani M, Zarei O, Alizadeh R, Masjedian F, Zarnani AH (2011). Extraction, Purification and Characterization of Lipopolysaccharide from *Escherichia coli* and *Salmonella typhi*. Avicenna J Med Biotechnol.

[31] Coligan JE (2003). Current Protocols in Immunology.

[32] Kimura M, Imaoka K, Suzuki M, Kamiyama T, Yamada A (2008). Evaluation of a microplate agglutination test (MAT) for serological diagnosis of canine brucellosis. J Vet Med Sci.

