# Supplementary material for: Development of an improved competitive ELISA based on a monoclonal antibody against lipopolysaccharide for the detection of bovine brucellosis
Source: BMC Vet Res. 2015 May 21;11:118. doi: 10.1186/s12917-015-0436-3 (PMC4438517; doi:10.1186/s12917-015-0436-3)
Supplement: Additional file 1: — Epitope mapping of MAbs (C/Y(M = A)) on additivity test. The additivity index (AI) value were calculated. The value below 50 indicated that the MAbs tested recognized the same epitope. [file 12917_2015_436_MOESM1_ESM.docx]

**Table S1 Epitope mapping of MAbs (C/Y(M=A)) on additivity test**

| MAbs | 4C3 | 4H7 | 6F2 | II5G1 | II4D11 | 6B8 |
| --- | --- | --- | --- | --- | --- | --- |
| 4C3 | / | 7 | 3 | -4 | 19 | 2 |
| 4H7 | 0 ^a^ | / | 7 | 6 | 20 | 29 |
| 6F2 | 1 | 1 | / | -1 | 33 | 17 |
| II5G1 | -4 | 6 | -1 | / | 10 | 11 |
| II4D11 | 19 | 20 | 33 | 10 | / | 21 |
| 6B8 | 2 | 29 | 17 | 11 | 21 | / |

^a^ The additivity index (AI) value were calculated using the fomula: AI=[2A_1+2_/(A_1_+A_2_)-1]×100%, where A_1_, A_2_ and A_1+2_ are the absorptions with the first antibody alone, the second antibody alone and the two antibldies together respectively (Bertrand et al. 1983). The MAbs were diluted to saturate the coated whole-cell antigen. The lowest AI value of 50 was defined as the threshold for evaluating epitope relatedness (Portner et al. 1987).
