# Supplementary material for: Development of an improved competitive ELISA based on a monoclonal antibody against lipopolysaccharide for the detection of bovine brucellosis
Source: BMC Vet Res. 2015 May 21;11:118. doi: 10.1186/s12917-015-0436-3 (PMC4438517; doi:10.1186/s12917-015-0436-3)
Supplement: Additional file 2: — Detection results of four serological diagnosis. The detail detection results of 110 bovine sera samples and 6 hyperimmune antisera in four serological diagnosis were shown in the table. [file 12917_2015_436_MOESM2_ESM.docx]

**Table S2 Detection results of four serological diagnosis**

| Serum sample | cELISA  PI≥40% | Svanova ELISA PI≥30% | RBT^a^ | MAT^b^ |
| --- | --- | --- | --- | --- |
| 3589 | 96.7 | 39.7 | + | 3200 |
| 3629 | 95.3 | 99.1 | ++ | 1600 |
| 3807 | 94.7 | 98.3 | +++ | 1600 |
| 6083 | 96.2 | 98.8 | + | 1600 |
| 38071 | 93.2 | 99.3 | ++++ | 1600 |
| 0063 | 95.3 | 99.4 | +++ | 800 |
| 3523 | 82.6 | 98.2 | ++ | 800 |
| 3524 | 95.4 | 98.1 | ++ | 800 |
| 3539 | 87.9 | 98.8 | ++ | 800 |
| 3542 | 95.1 | 97.6 | ++ | 800 |
| 3578 | 95.2 | 37.3 | +++ | 800 |
| 3836 | 95.4 | 99.3 | ++ | 800 |
| 3852 | 94.9 | 99.4 | ++ | 800 |
| 6049 | 94.9 | 98.7 | ++ | 800 |
| 6073 | 92.7 | 99.4 | + | 800 |
| 3452 | 88.3 | 98.9 | + | 400 |
| 3465 | 83.9 | 99 | ++ | 400 |
| 3534 | 95.5 | 99.4 | ++ | 400 |
| 3587 | 76.8 | 98.6 | +++ | 400 |
| 3685 | 95 | 98.8 | + | 400 |
| 3724 | 62.2 | 99 | ++ | 400 |
| 3848 | 86.9 | 98.6 | + | 400 |
| 3879 | 66.6 | 99.3 | ++ | 400 |
| 3970 | 94.5 | 99.5 | ++ | 400 |
| 6014 | 94.4 | 99.1 | +++ | 400 |
| 6531 | 42.1 | 99.2 | +++ | 400 |
| 6557 | 65.2 | 64.5 | +++ | 400 |
| 3826 | 53.8 | 98.4 | +++ | 200 |
| 3855 | 82.4 | 99.1 | ++ | 200 |
| 3890 | 89 | 99.2 | ++ | 200 |
| 3895 | 69.4 | 97.9 | ++ | 200 |
| 4020 | 90.8 | 97.4 | ++ | 200 |
| 6302 | 92.4 | 90.9 | ++ | 200 |
| 11015 | 55.3 | 67.6 | ++ | 200 |
| 3068 | 50.4 | 93.6 | ++ | 100 |
| 3647 | 65.4 | 94 | +++ | 100 |
| 3649 | 40.1 | 26.9 | +++ | 100 |
| 3657 | 78.2 | 99.2 | + | 100 |
| 3662 | 83.1 | 73.5 | ++ | 100 |
| 3711 | 80.2 | 97.5 | + | 100 |
| 3862 | 54.5 | 22.9 | + | 100 |
| 3866 | 70.7 | 29 | + | 100 |
| 6062 | 42 | 43.9 | ++ | 100 |
| 6069 | 68 | 94.6 | ++ | 100 |
| 6124 | 60 | 29.5 | + | 100 |
| 10012 | 73.4 | 35.2 | ++ | 100 |
| 36572 | 44.7 | 99.3 | + | 100 |
| 3553 | 50.8 | 19.4 | ++ | 50 |
| 3614 | 53.5 | 69.8 | ++ | 50 |
| 3713 | 46.7 | 49.1 | + | 50 |
| 3758 | 69.9 | 99.3 | ++ | 50 |
| 3834 | 52.4 | 94 | ++ | 50 |
| 3866 | 45.3 | 48.2 | + | 50 |
| 3902 | 52.2 | 9.9 | + | 50 |
| 6062 | 42 | 43.9 | ++ | 50 |
| 6091 | 40.4 | 0.5 | + | 50 |
| 10051 | 66.9 | 77.7 | ++ | 50 |
| 3502 | 15.7 | -14.9 | + | 25 |
| 3682 | 51.7 | 8.3 | + | 25 |
| 3766 | 6.1 | -3.3 | - | 25 |
| 4023 | 34.7 | 9.9 | ++ | 25 |
| 6067 | 56 | 22.4 | + | 25 |
| 6089 | 4.4 | 18.4 | ++ | 25 |
| 6119 | 42.7 | 38.6 | ++ | 25 |
| 11001 | 28.4 | 6.9 | ++ | 25 |
| 11003 | 34.8 | 58.5 | + | 25 |
| 12008 | 13.1 | -21.9 | + | 25 |
| 12047 | 24.6 | -19.2 | - | 25 |
| 3306 | 12.7 | -18.5 | - | - |
| 3308 | 2.9 | -19.4 | - | - |
| 3314 | 0.1 | 3 | - | - |
| 3763 | 9.9 | 4.9 | + | - |
| 3994 | 29.4 | 2.6 | + | - |
| 6021 | 6.6 | 6.7 | + | - |
| 6092 | 27 | -8.9 | - | - |
| 6146 | 20.8 | 2.1 | - | - |
| 6159 | 27 | 10.2 | - | - |
| 10005 | 17.1 | 15.5 | - | - |
| 10022 | 22 | -44.3 | - | - |
| 10024 | 2.1 | -57.6 | - | - |
| 10089 | 16.5 | 34.3 | - | - |
| 11015 | 25.1 | -23.8 | - | - |
| 11025 | 12.4 | -20.9 | - | - |
| 11030 | 20.9 | 27.5 | - | - |
| 11033 | 29.4 | 16.8 | - | - |
| 11045 | 20 | 2.9 | - | - |
| 11047 | 23.7 | 15.9 | - | - |
| 11081 | 28 | 17.2 | - | - |
| 11088 | 5.8 | 7.1 | - | - |
| 11093 | 13.4 | 6.5 | - | - |
| 11101 | 14.8 | 1.3 | - | - |
| 12003 | 19.6 | 13.1 | - | - |
| 12010 | 19.2 | 16.5 | - | - |
| 12013 | 8.3 | 10.4 | - | - |
| 12015 | 26.2 | 5.6 | - | - |
| 12023 | 26.9 | -1.4 | - | - |
| 12024 | 11.1 | 4.9 | - | - |
| 12031 | 28.4 | -6 | - | - |
| 12034 | 13.7 | -4.8 | - | - |
| 12035 | 18.8 | -20.8 | - | - |
| 12042 | 3.3 | -10.4 | - | - |
| 12043 | 21.4 | 12.9 | - | - |
| 12044 | 22.1 | 7.7 | - | - |
| 12049 | 18.8 | -16.3 | - | - |
| 12053 | 24.9 | 21.6 | - | - |
| 12059 | 20.9 | 6.4 | - | - |
| 12061 | 10.6 | -6.5 | - | - |
| 12063 | 28.2 | 18.7 | - | - |
| 12064 | 10.5 | 8.5 | - | - |
| 120025 | 16.7 | 2.7 | - | - |
| *E. coli* O157 | 8.2 | -17.9 | - | - |
| *O. anthropic* | 4.4 | -3.8 | - | - |
| *F. tularensis* LVS | 6.0 | 8.6 | - | - |
| *S. gallinarum* | 18.4 | 10.7 | - | - |
| *Y. enterocolitica* O:9 | 8.9 | 61.5 | ++ | 25 |
| *P. multocida* | 0.1 | -3.6 | - | - |

^a^ “-”indicates no reaction and resulted as negative; numbers of “+” indicate response intensity, ie. “++++” stands for agglutinate slice of a big or small particles; “+++” stands for a significant agglutinate slice; “++” stands for visible agglutinate slice; “+” stands for only a small amount of granular material and turbid liquid.

^b^ The titers were expressed as a reciprocal of the highest dilution of sera showing completely agglutination.
